# Effects of the amount of practice and time interval between practice sessions on the retention of internal models

**DOI:** 10.1371/journal.pone.0215331

**Published:** 2019-04-16

**Authors:** Chiharu Yamada, Yoshihiro Itaguchi, Kazuyoshi Fukuzawa

**Affiliations:** 1 Graduate School of Letters, Arts and Sciences, Waseda University, Tokyo, Japan; 2 Research Fellow of Japan Society for the Promotion of Science, Tokyo, Japan; 3 Faculty of Informatics, Shizuoka University, Hamamatsu, Japan; 4 School of Humanities and Social Sciences, Waseda University, Tokyo, Japan; Carl von Ossietzky Universitat Oldenburg, GERMANY

## Abstract

The amount of practice and time interval between practice sessions are important factors that influence motor learning efficiency. Here, we aimed to reveal the relationship between the retention and consolidation of a new internal model, and the amount of practice and time interval between practice sessions. We employed a visuomotor rotation tracking task to test the hypotheses that (1) a new internal model consolidates owing to extensive practice after reaching a task performance plateau and (2) a longer time interval between practice sessions makes it difficult to activate a new internal model. The participants were assigned to one of the four groups that differed in terms of the amount of practice and the time interval between practice sessions. They performed a tracking task in which they experienced 120° clockwise visuomotor rotation and were required to track a moving target on a computer display using a mouse cursor. To evaluate the retention and consolidation of a new internal model, we calculated the aftereffects and savings as measures of motor learning. To the best our knowledge, this is the first study to manipulate both the amount of practice and the time interval between practice sessions simultaneously in one experiment using a visuomotor tracking task. Our results support the previously reported idea that extensive practice is necessary for the consolidation of a new internal model.

## Introduction

The efficiency of human motor learning is significantly enhanced by minimizing the amount of practice, while maximizing the time interval between practice sessions without losing what is learned. Various studies have been conducted to model the optimal method of practice for maximizing motor learning performance [1]. For example, the effects of practice conditions, such as contextual interference and knowledge of results [2–4] and physical fatigue [5] on performance have been investigated. Besides these factors, we focus on the practice schedule, i.e., the duration of practice and the time interval between practice sessions. For people who try to learn or improve a new motor skill, a critical problem is how long they should practice in a day and how many days of rest they are allowed between sessions to maintain their skills. Therefore, to establish a method for efficient motor learning, it is necessary to determine the effects of the amount of practice and the time interval between practice sessions on motor learning.

### Effect of amount and time interval of practice

In terms of the amount of practice, neurophysiological studies have suggested that extensive practice is important for the consolidation and retention of motor memory [6]. In addition, imaging studies on human motor sequence learning and recent studies on human visuomotor adaptation have shown that saturation learning caused by extensive practice facilitates the retention of newly acquired motor skills [7–10]. For efficient motor learning, the time interval between practice sessions should not be too long or too short. Passage of time is one of the main factors that decays motor memory [8, 11–13]. However, some studies have reported that a certain time interval facilitates the consolidation of motor memory [14–16]. Shadmehr and his colleagues [14–16] suggested that time passage is necessary to reduce task-irrelevant motor commands and stabilize motor memory. Therefore, to avoid wasting time and effort, it is necessary to determine the optimal time interval.

Krakauer, Ghez [8] suggested that consolidation for visuomotor learning becomes more resistant to retrograde interference when the amount of initial learning is increased, and that a longer time interval causes time decay of consolidated motor memory. In a series of experiments, they used a reaching task with visuomotor rotation to test the retrograde interference effect. They manipulated the time interval between the initial rotation learning and counter-rotation learning (5 min vs 24 h). The time interval between the counter-rotation learning and rotation re-learning was 24 h or 48 h. The amount of initial learning was 264 trials or 528 trials. The results showed that the amount of the initial practice contributed to a stronger resistance to interference. In addition, they indicated that a longer time interval caused time decay even in consolidated motor memory.

Trempe and Proteau [9] showed that a 24-h interval after extensive practice facilitates internal model consolidation. They performed an experiment involving a reaching task, in which they manipulated the amount of practice in the first session (24 trials vs. 144 trials) and the time interval between the first session and re-learning session (10 min vs. 24 h). The results of the experiment showed that when the participants performed 24 trials in the first session, their performance in the re-learning session decreased to the baseline level despite the different time interval conditions. In contrast, when the participants performed 144 trials in the first session, the 24-h-interval group showed more persistent aftereffects than the 10-min-interval group. From these results, it was concluded that performance improvement to a certain level in the first session leads to internal model consolidation after 24 h.

Although these previous studies investigated the consolidation of motor memory by manipulating the amount of the practice or time interval between practice sessions, the interaction between them in long-term motor learning has not been directly addressed. Trempe and Proteau [9] focused on the difference between short-term retention (10 min) and long-term retention (24 h) of motor memory after the first practice session. Further, in a previous study of considerable importance, Krakauer, Ghez [8] performed several experiments with different amounts of practice and time intervals between practice sessions; however, they did not directly investigate the interaction between the time interval until the re-learning session and the amount of practice in the initial session. Thus, regarding the amount of practice and time interval between practice sessions, it remains unclear how these factors affect the acquisition and long-term retention of a new internal model.

The current study aimed to determine the effect of the amount of practice and long-term intervals between practice sessions on the retention and consolidation of an internal model. We assumed that motor learning and consolidation within the time frame of a day is important for the optimization of the practice schedule in motor skill learning. Therefore, we scheduled an experiment based on a longer span of time than that used by Trempe and Proteau [9]. We manipulated the amount of practice on Day 1 (20 sessions vs. 10 sessions) and the time interval between practice sessions (24 h vs. 48 h). The objective of the present study was to determine the relationship between motor learning and amount of practice and time intervals between practice sessions.

### Internal model of visuomotor rotation

The acquisition of motor skills can be interpreted as that one acquires a new internal model for the skill. An internal model is a neural mechanism in the central nervous system (CNS) that can mimic and simulate the behavior of the sensorimotor system and objects in the real world, that is, the external environment outside of our body [17]. Using this model, one can predict sensory feedback as the consequences of movements by using efference copies of motor commands [18, 19]. Assuming that internal models exist in the CNS, we can explain how humans solve the problem regarding time delay during visual feedback in motor control to achieve fast and smooth movement and to flexibly adapt to changes in the real world [20–22]. Neurophysiological and imaging studies have suggested that the cerebellum acquires internal models by using error signals that are the differences between the predicted and actual sensory consequences [19, 23–25]. These studies suggested that the cortico-cerebellar network is the neural substrates involved in internal models for motor production and motor control [20, 23, 26].

### Aftereffects and savings

The degree of motor learning progression is assessed based on aftereffects and savings. These two measures were calculated from the task performance, which is evaluated as the distance between the target and the cursor. Aftereffects refers to a rapid increase in error during a motor learning task, which is observed when the external environment changes after the participants adapt to the previous environment. For example, in visuomotor adaptation tasks, the most important information about the external environment is regarding how the mouse cursor moves according to the movement of the participants’ hand; in other words, it describes how the movement direction of the mouse cursor is perturbed by visuomotor rotation. When participants return to a normal environment (rotation is OFF) after they have adapted to a novel environment (rotation is ON), error increases even they execute the task under the normal environment. This phenomenon is called “aftereffects”. Usually, aftereffects vanish relatively quickly; that is, the error in the normal environment changes back to the baseline in a short while. It is a common measure for visuomotor adaptation [8, 27–31], and it is considered as a sign of acquisition of a new internal model [32]. Predictive feedforward control based on internal models is necessary to achieve fast and smooth movement that is required in a tracking task [33]. In human motor learning, feedback control is dominant until a new internal model is acquired, while feedforward control becomes dominant once the new internal model consolidates [17, 20]. Therefore, we evaluated aftereffects to examine whether the participants acquired a new internal model for a novel environment.

Savings is defined as faster re-learning of a novel environment compared to the initial learning. [34, 35]. For example, when participants are exposed to a novel environment (rotation is ON) in the visuomotor adaptation tasks twice with some time interval in between, error decreases faster in the second exposure (re-learning) than in the first exposure (initial learning). This improvement in the efficiency of motor learning is called savings. The calculations of savings differ among studies [36], but they are consistent with each other in that savings is considered as an index of recalling the motor memory of visuomotor rotation that has already been learned [8, 28, 37]. In the present study, we assessed savings to test how the amount of initial training and time interval between practice sessions affected the retention of motor memory.

To determine the effects of the amount of practice and the time interval between practice sessions on the retention and consolidation of a new internal model, the present study investigated the following two hypotheses: (1) A new internal model consolidates owing to extensive practice after reaching a task performance plateau. (2) As the time interval between practice sessions is extended, it prevents the new internal model from being activated.

### Implicit learning and visuomotor tracking task

In the present study, we conducted a tracking task with 120° clockwise visuomotor rotation for two days based on the work of Imamizu, Miyauchi [24] because we assumed that the contributions of explicit strategies are less in the tracking task compared to the reaching task. Previous studies on visuomotor adaptation commonly involved a reaching task that required the participants repeatedly to execute short reaching movements from a start point to a target [36, 38–42]. In contrast, the tracking task we conducted requires intuitive motor control of participants to keep tracking a randomly moving target with a cursor.

We assumed that with continuous movement in the tracking task, participants are less likely to use explicit strategies compared to the probability of the reaching task because continuous movement in the tracking task does not allow the participants to take time for considering how to reduce errors during trials. While the target of the tracking task keeps moving and the optimal movement to reduce errors changes from moment to moment, that of the reaching task is static during the movement, likely enabling participants to develop a solution for error reduction [43, 44]. In the learning session with visuomotor rotation, it is more complicated to move a cursor in a direction that compensates for the imposed rotation in the tracking task than in the reaching task. Previous studies have reported that a tracking task facilitates implicit learning as it requires continuous and omnidirectional movements [45, 46]. Although it would be indirect evidence regarding the implicit nature of tracking-based adaptation, rotation-induced errors in general decrease slowly in tracking tasks compared to reaching tasks [24, 43, 44]. In addition, recent studies reported a relatively large contribution of explicit strategies in a reaching task involving visuomotor rotation [47–50]. Although we do not exclude the possibility that the tracking task involves explicit components, to decrease the influences of explicit learning and investigate implicit learning, we used the tracking task. Note that this does not mean that the tracking task can eliminate all explicit strategies because as Taylor, Krakauer [51] indicated, an explicit component is inevitably involved with motor adaptation tasks.

## Material and methods

### Participants

The study and consent procedure were approved by the Ethics Committee on Human Research of Waseda University. Forty right-handed university students (20 males, 20 females; mean age: 20.8±1.9 years) provided informed consent and performed a tracking task. All the participants were neurologically healthy and unaware of the purpose of the study and the experimental task. The participants were assigned to one of four experimental condition groups that differed in terms of the amount of practice on Day 1 and the time interval between Day 1 and Day 2. We controlled possible circadian effects within participants by carrying out the task on Day 1 and Day 2 in the same period of time. The participants were told to get a good night’s sleep and to avoid all forms of caffeine and alcohol for the duration of their participation in the experiment.

### Experimental setup

**Fig 1:**
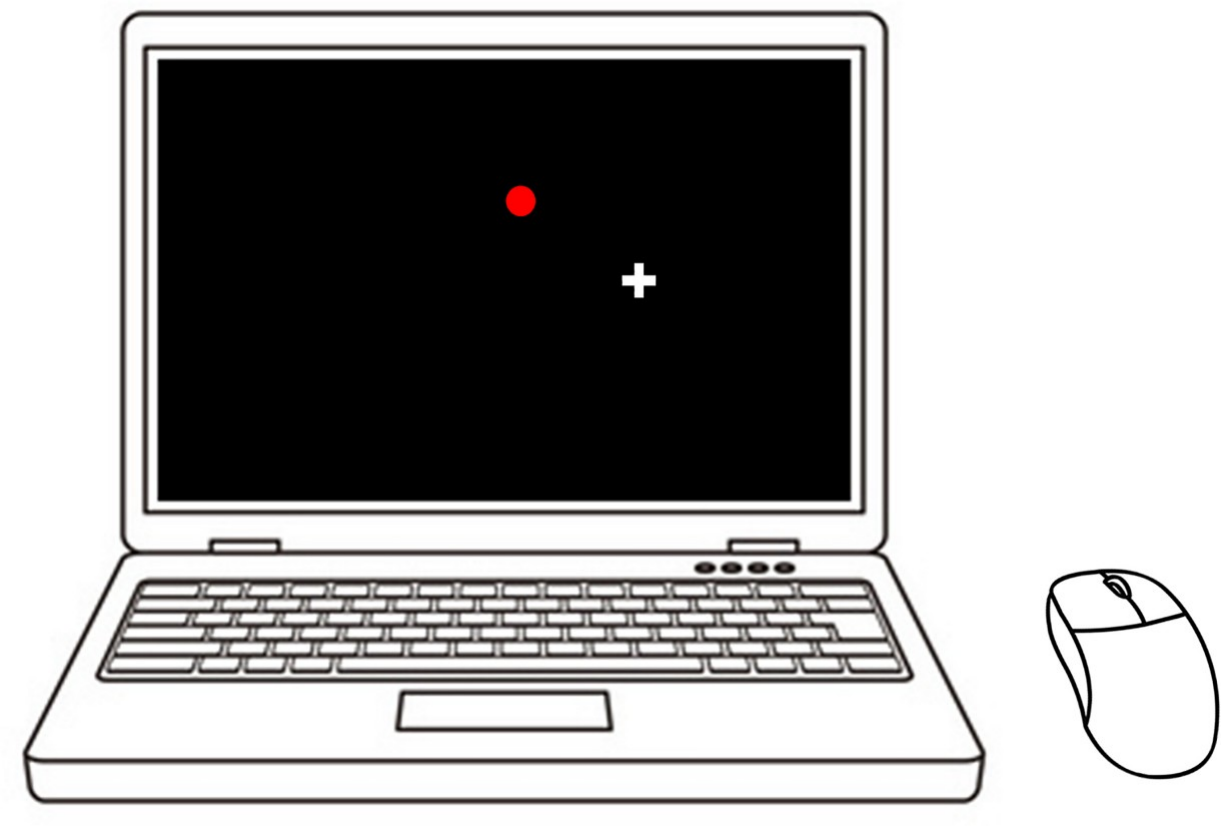
Experimental apparatus. The participants sat on a height-adjustable chair in front of a desk and performed a tracking task. All the participants used the same computer mouse and laptop computer (Dell, Inspiron 13” screen) placed 60 cm in front of them.

Fig 1 shows the experimental apparatus. The participants sat on a height-adjustable chair in front of a desk and performed a tracking task. All the participants used the same mouse and laptop computer (13” Dell Inspiron) placed 60 cm in front of them. The participants moved the mouse on the surface of the horizontal desk with their right hand, and they were instructed to keep the mouse straight when they moved it. The experimenter observed the participants from a separate room during the task to check whether they carried out the task according to the instructions.

### Tracking task

The tracking task was created on the basis of the work of Imamizu, Miyauchi [24]. The participants performed the task on two days, separated by an interval of 24 h or 48 h. They were instructed to track a moving ball (2 mm in diameter) using a cross-hair cursor (2 mm). The target ball moved at an average speed of 11 cm/s. The trajectory of the target was the sum of two waves. The trajectories were generated randomly from sine waves with two different frequencies along the x and y axes. The target started its movement from the center of the display in every trial. Each trial took 30 s and there was a 5 sec break between the trials. One session involved four trials.

**Fig 2:**
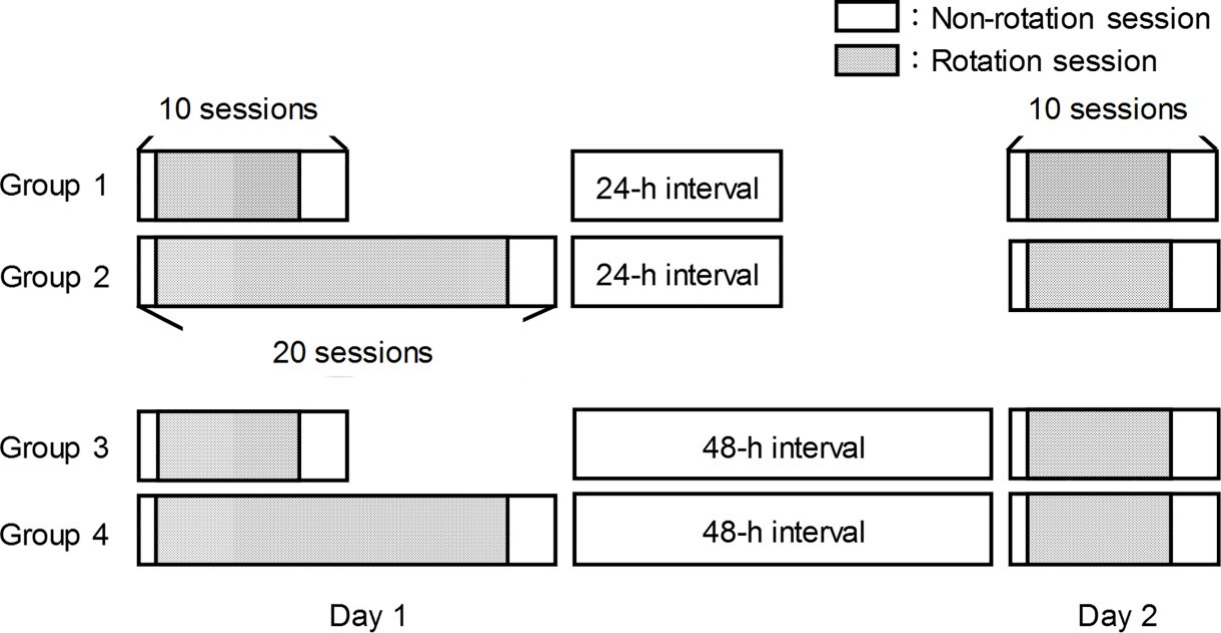
Training schedule. The participants were assigned to one of four groups that differed in terms of the amount of practice on Day 1 and the time interval between Day 1 and Day 2. The gray parts of the bars represent rotation sessions, and the white ones represent non-rotation sessions. Groups 1 and 2 performed the task twice with a 24-h interval, while Groups 3 and 4 performed it twice with a 48-h interval. Groups 1 and 3 performed 10 sessions on Day 1, whereas Groups 2 and 4 performed 20 sessions on Day 1.

The practice schedule is shown in Fig 2. The task consisted of three sessions: the baseline session, practice session, and testing session. In the baseline and testing sessions, visuomotor rotation was not introduced along the movement direction of the mouse cursor; therefore, the relationship between the movement direction of the mouse and the mouse cursor was normal. In the practice session, the visual feedback of the mouse cursor was rotated 120° clockwise. There were four experimental conditions that differed in terms of the amount of practice on Day 1 and the time interval between Day 1 and Day 2. On Day 1, two groups (Group 1, Group 3) performed 20 sessions including one baseline session (rotation is OFF), 16 practice sessions (rotation is ON), and three testing sessions (rotation is OFF). The other two groups (Group 2, Group 4) performed 10 sessions on Day 1, including one baseline session, six practice sessions, and three testing sessions. The cursor was rotated similar to the practice sessions for Groups 1 and 3. As for the time interval between practices, two groups (Group1, Group 2) performed the second experimental session 24 h later, while the others (Group 3, Group 4) did so 48 h later. On Day 2, all the groups performed 10 sessions including one baseline session, six practice sessions, and three testing sessions. The 10-session practice took approximately 30 min, and the 20-session practice took approximately 60 min.

The target ball was colored green in the non-rotation condition and red when the cursor was rotated 120° clockwise. Prior to the experiment, the participants were instructed to concentrate on tracking the target, and not to move the cursor in other ways. In addition, they were told that the path of the target and the cursor movement were controlled in the experiment, and that they might find the experiment unusual. The tracking task was written in MATLAB, using the Psychophysics Toolbox extensions [52, 53].

### Data analysis

All the trajectories of the ball and cursor were recorded using MATLAB at 60 Hz. The tracking error was calculated as the Euclidian distance between the target and cursor. Based on this error calculation, the aftereffects and savings were calculated as measures of visuomotor adaptation and motor learning, respectively. In the present study, the difference between the first baseline session and first testing session was considered as the aftereffects. For calculating the aftereffects, we considered the positional error in the initial two seconds of each trial. We did not use the last two seconds because the error in that period was likely influenced by the tracking performance before the period, which included a large amount of feedback influence. It was observed that at least in the tracking task, the distance error of the first few seconds well reflected a feedforward component and stable rather than the initial directional error [43]. In addition, the savings was calculated as the percent decrease in error from the second session to the seventh session from Day 1 to Day 2.

### Statistical testing

To test if the adaptation reached a learning plateau, we performed a paired t-test to compare errors between the last two sessions within each group. In terms of the aftereffects, we performed a three-way, mixed measures ANOVA to test whether the change in error from the last practice session to the next session differed between the four groups that varied in terms of the amount of practice and the time interval between practice sessions. In terms of savings, we performed a two-way repeated measures ANOVA to test whether the decrease rate of the error changed from Day 1 to Day 2. The statistical test used in the present study followed that used in previous studies [24, 30, 43–46].

## Results

**Fig 3:**
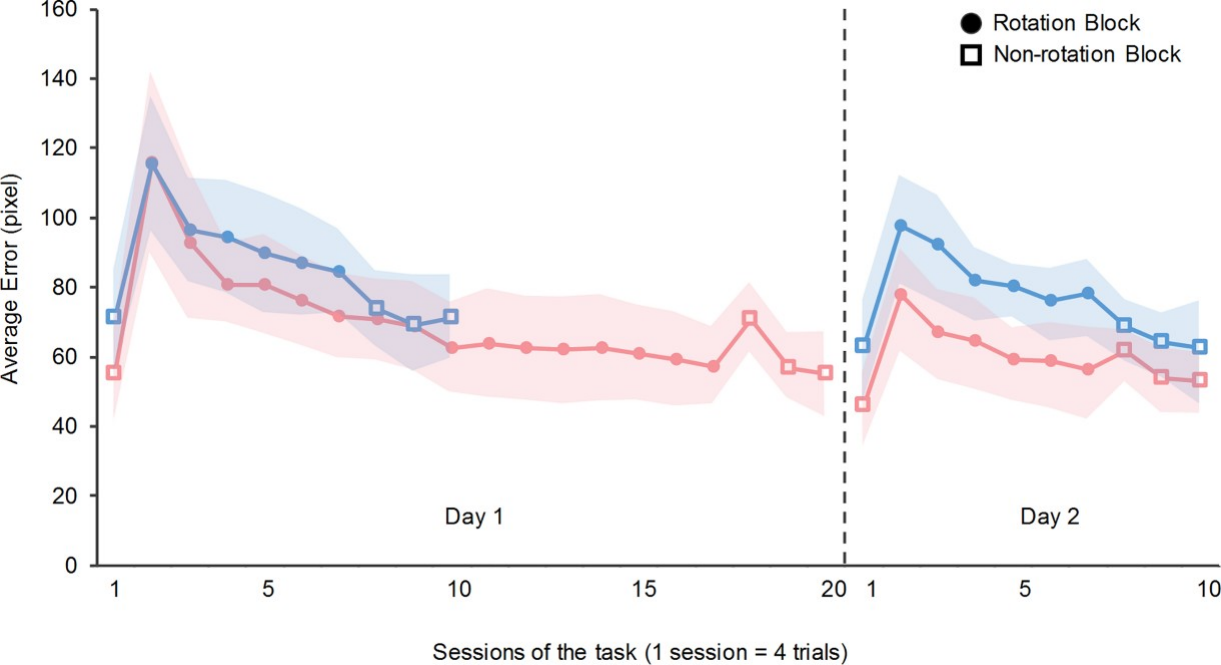
Task performance of Group 1 and Group 2 (24-h-interval groups). The horizontal axis represents the number of sessions of the tracking task, and the vertical axis represents the average error. The dashed line separates the results of Day 1 and Day 2. The blue line represents Group 1 (participants who performed 10 sessions on Day 1). The pink line represents Group 2 (participants who performed 20 sessions on Day 1). The open squares represent non-rotation sessions, and the filled circles represent rotation sessions. The background shadow shows the standard deviation of each group. The error increased in the second session on Day 1 in both the groups, after which it decreased.

**Fig 4:**
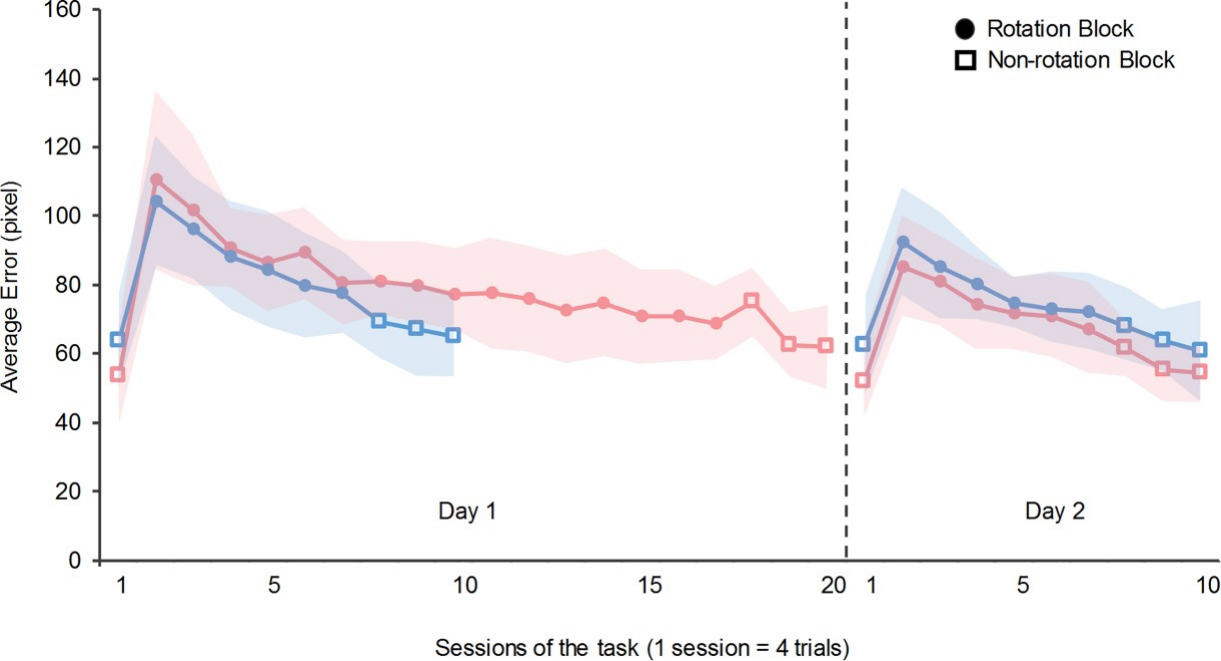
Task performance of Group 3 and Group 4 (48-h-interval groups). The horizontal axis represents the number of sessions of the tracking task, and the vertical axis represents the average error. The dashed line separates the results of Day 1 and Day 2. The blue line represents Group 3 (participants who performed 10 sessions on Day 1). The pink line represents Group 4 (participants who performed 20 sessions on Day 1). The open squares represent non-rotation sessions, and the filled circles represent rotation sessions. The background shadow shows the standard deviation of each group. As with the 24-h-interval groups, the error increased in the second session on Day 1 in both the groups, after which it decreased.

We calculated the errors in the tracking task for two days to investigate the effect of the amount of practice and time interval between practice sessions on the retention and consolidation of a new internal model. Although all participants noticed the rotation of the cursor in the practice sessions, they could not describe the rotation. Fig 3 shows the changes in the errors of the 24-h-interval groups, and Fig 4 shows those of the 48-h-interval groups. The horizontal axis represents the number of sessions, and the vertical axis represents the task errors.

The errors increased in the second session on Day 1 and decreased after the second session. From the 2nd session to the 17th session, the tracking errors decreased by 50.1±10.9% in Group 1 and 37.8±13.6% in Group 3. From the 2nd session to the 7th session, the error decreased by 26.9±12.0% in Group 2 and 25.5±13.64% in Group 4. In addition, to confirm whether the tracking errors reached a plateau at the end of the practice sessions, we performed a paired *t*- test for the errors in the last two practice sessions for each group. The results showed that there were no significant differences between the last two sessions in all groups (*t*(18) = 0.36, *p* = 0.72; *t*(18) = 0.32, *p* = 0.76; *t*(18) = 0.29, *p* = 0.77; *t*(18) = 0.27, and *p* = 0.79).

**Fig 5:**
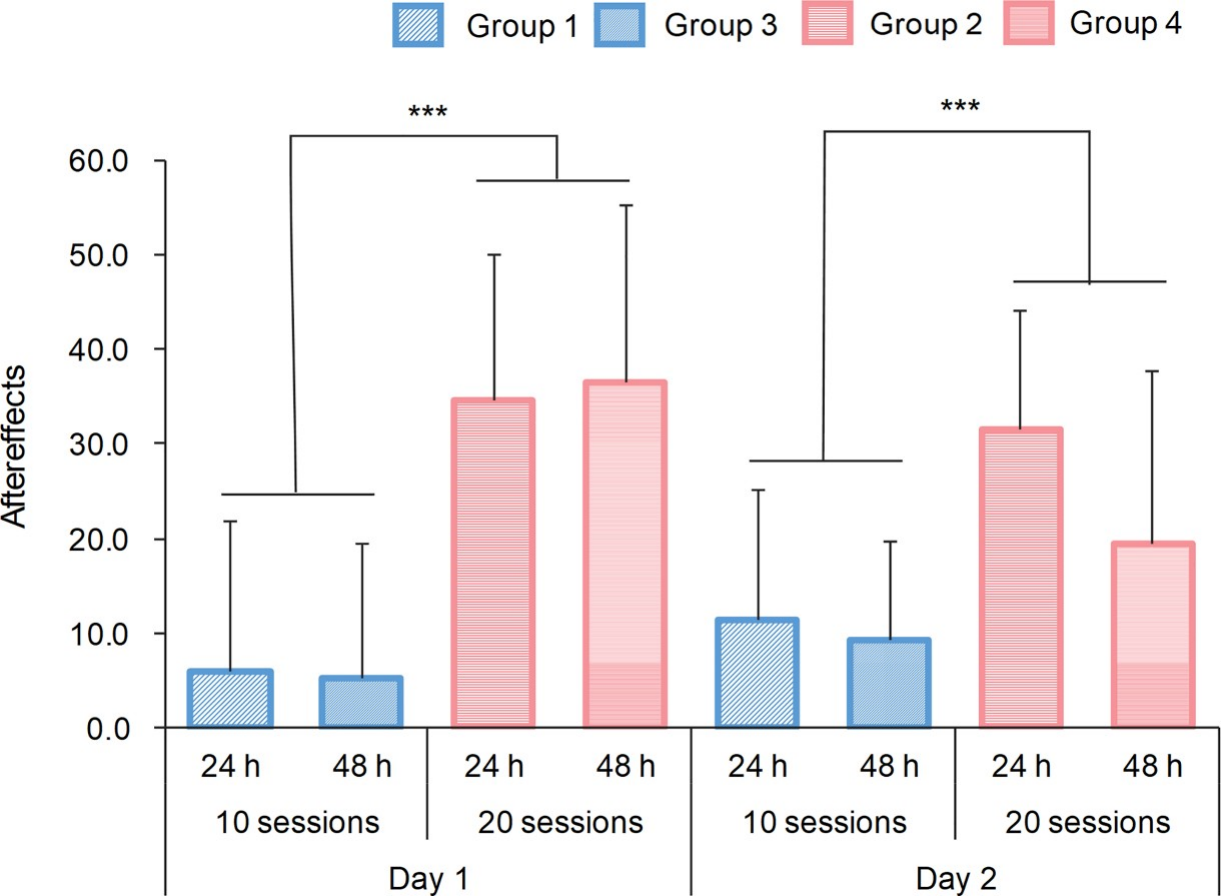
Aftereffects on Day 1 and Day 2. The vertical axis represents the difference between the last rotation session and the first non-rotation session (baseline). A mixed-design ANOVA revealed a statistically significant interaction between the day of training and the amount of training (*F*(1,36) = 8.14, *p* < 0.001, *η*_*p*_^2^ = 0.18) and the main effects of the amount of training on Day 1 (*F*(1,36) = 31.85, *p* < 0.001, *η*_*p*_^2^ = 0.47). A post-hoc test revealed the simple main effect of the amount of training on Day 1 and Day 2 (*F*(1,36) = 34.52, *p* < 0.001, *η*_*p*_^2^ = 0.49; *F*(1,36) = 11.78, *p* < 0.01, *η*_*p*_^2^ = 0.25) and that of the day of practice in the 20-session groups (*F*(1,18) = 6.87, *p* < 0.05; *η*_*p*_^2^ = 0.28). The aftereffects marked with asterisks were significantly higher in the 20-session practice groups than in the 10-session practice groups (all *p*s < .001).

The aftereffects differed depending on the amount of practice. Fig 5 shows the results. A three-way mixed ANOVA with the day of practice (Day 1, Day 2) as the within-subject factor and the time interval (24 h vs. 48 h) and the amount of practice on Day 1 (20 sessions vs. 10 sessions) as the between-subject factors revealed a statistically significant interaction between the day of practice and the amount of practice (*F*(1,36) = 8.14, *p* < 0. 001, *η*_*p*_^2^ = 0.18) and the main effect of the amount of practice on Day 1 (*F*(1,36) = 31.85, *p* < 0. 001, *η*_*p*_^2^ = 0.47). A post-hoc test revealed the simple main effect of the amount of practice on Day 1 and Day 2 and that of the day of practice on the 20-session groups (*F*(1,36) = 34.52, *p* < 0.001, *η*_*p*_^2^ = 0.49; *F*(1,36) = 11.78, *p* < 0.01, *η*_*p*_^2^ = 0.25; *F*(1,18) = 6.87, *p* < 0.05, *η*_*p*_^2^ = 0.28).

In addition, we conducted two-way mixed ANOVA for each time interval condition. The results showed that in the 48-h-interval groups, there was a statistically significant interaction between the amount of practice and the day of practice, and a significant main effect of the amount of practice (*F*(1,18) = 9.84, *p* < 0.001, *η*_*p*_^2^ = 0.35; *F*(1,18) = 11.34, *p* < 0.01, *η*_*p*_^2^ = 0.39). A post-hoc test revealed that the simple main effects of the amount of practice on Day 1 and the day of practice on the 20-session practice condition were significant (*F*(1,18) = 17.85, *p* < 0.001, *η*_*p*_^2^ = 0.50; *F*(1,18) = 10.82, *p* < 0.01, *η*_*p*_^2^ = 0.55). In summary, in the 48-h-interval groups, the aftereffects in the group of participants who performed 20 sessions of the task on Day 2 was smaller than that on Day 1.

**Fig 6:**
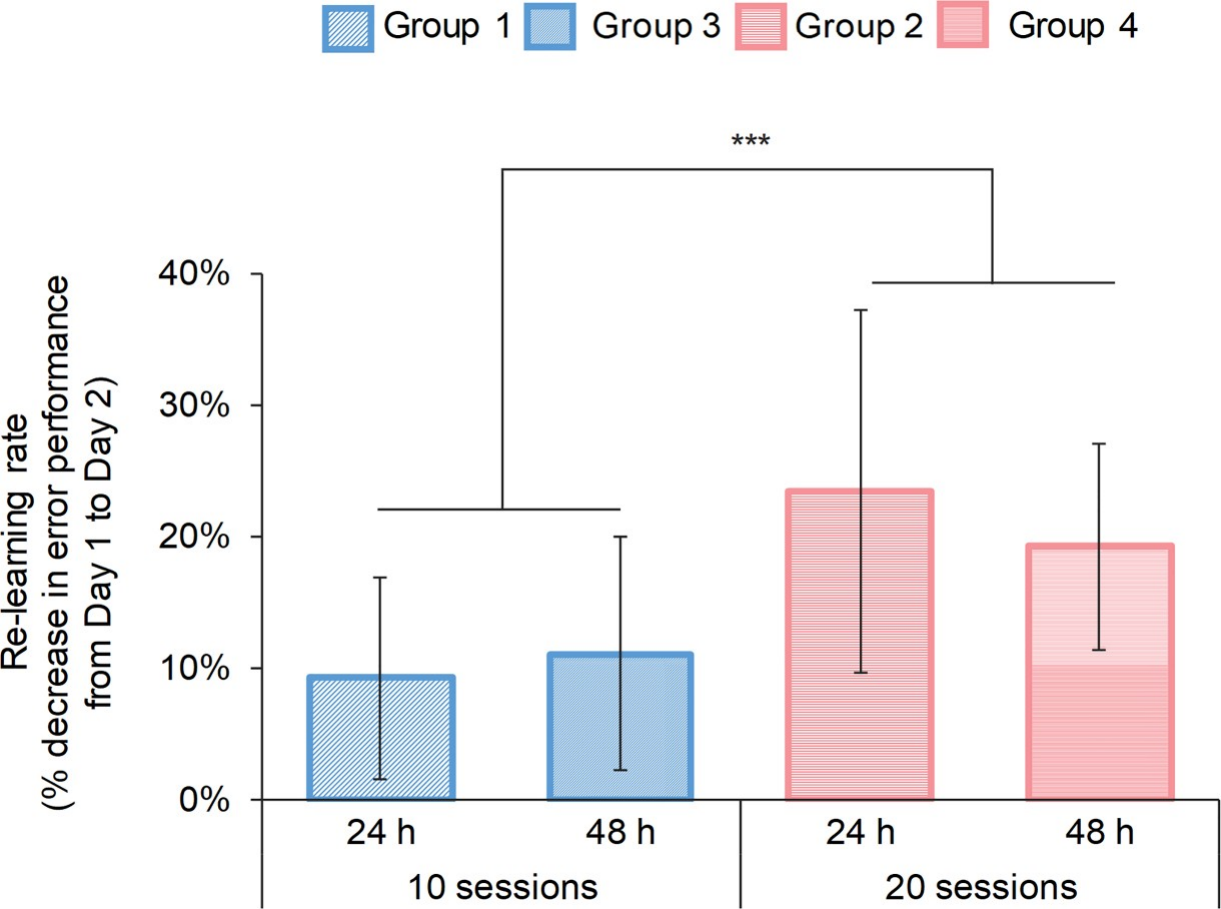
Savings in the experimental groups. The vertical axis represents savings. The savings of the 20-session groups (Group 1, Group 3) was greater than that of the 10-session groups (Group 2, Group 4). A two-way ANOVA with the time interval (24 h, 48 h) and the amount of training on Day 1 (20 sessions, 10 sessions) as between-subject factors revealed the main effect of the amount of training (*F*(1,36) = 12.89, *p* < 0.001, *η*_*p*_^2^ = 0.26). There was no significant interaction. The savings values marked with asterisks were significantly higher in the 20-session practice groups than in the 10-session practice groups (all *p*s < .001).

As for savings, the improvement in performance from Day 1 to Day 2 of the 20-session groups (Group 1, Group 3) was greater than that of the 10-session groups (Group 2, Group 4). Fig 6 shows the results. A two-way ANOVA with the time interval (24 h, 48 h) and the amount of practice on Day 1 (20 sessions, 10 sessions) as the between-subject factors revealed the main effect of the amount of practice(*F*(1,36) = 12.89, *p* < 0.001, *η*_*p*_^2^ = 0.26). There was no significant interaction.

Further, we calculated the off-line learning gains on which Trempe and Proteau [9] focused as an index of motor learning. The off-line gains were found to be 10.1 in Group 1, 13.9 in Group 2, 8.7 in Group 3, and 10.5 in Group 4. A two-way between-subject ANOVA showed neither significant main effects of the amount of practice or the time interval of practice, nor a significant interaction between these two factors (*F*(1,36) = 0.83, *n*.*s*., *η*_*p*_^2^ = 0.02; *F*(1,36) = 1.09, *n*.*s*., *η*_*p*_^2^ = 0.30; *F*(1,36) = 0.71, *n*.*s*., *η*_*p*_^2^ = 0.03).

## Discussion

The present study investigated the effect of the amount of practice and the time interval between practice sessions on the retention and consolidation of a new internal model. We hypothesized that (1) a new internal model consolidates owing to extended practice after reaching a task performance plateau and (2) a longer time interval between practice sessions prevents the new internal model from being activated. The results of the tracking task for two days revealed that the magnitude of the aftereffects and the savings changed depending on the amount of practice, while the time interval between practice sessions affected the aftereffects only when the participants practiced longer. Specifically, the aftereffects in the 20-session groups (Group 2 and Group 4) were greater than those in the 10-session groups (Group 1 and Group 3) on Day 1 and Day 2 regardless of the time interval between the experimental sessions. In the 48-h-interval condition, the aftereffects decreased on Day 2 compared to Day 1, where the participants performed 20 sessions of the task on Day 1 (Group 4). In addition, the savings were greater in the 20-session groups (Group 2 and Group 4) than in the 10-session groups (Group 1 and Group 3), regardless of the time interval. These results supported the first hypothesis, but not the second one. By using a tracking task, the present study revealed for the first time that additional practice after reaching a task performance plateau is important for the consolidation of a new internal model.

### Saturation learning and consolidation of internal models

The present results supported the first hypothesis; the consolidation of a new internal model is stabilized by extended practice after reaching a task performance plateau. In the present study, the amplitude of the aftereffects was greater in the two groups where the participants performed 20 practice sessions on Day 1 (Group 2 and Group 4) than in the other two groups where the participants performed 10 practice sessions on Day 1(Group 1 and Group 3). Note that the error of the tracking task in all four groups did not significantly decrease in the last two practice sessions with visuomotor rotation on Day 1. Although we cannot provide direct evidence to conclude that Groups 1 and 3 completely reached a learning plateau, the improvement in the performance in the present study did not statistically change in the last two practice sessions in all the groups. These results indicate that just a decrease in the error of the task performance to the baseline is insufficient for the retention and consolidation of a new internal model, and extended practice after reaching a performance plateau is important for the consolidation of motor learning.

From this point of view, the present study is consistent with previous studies that suggested the importance of saturation learning in the consolidation of motor memory through a reaching task [8, 9, 28, 54]. The results of previous studies that used tracking tasks [24, 55] also agree with this idea, although their experiments focused on other issues. In the present study, consistent with these studies, larger aftereffects were observed in the two groups where the participants kept practicing after their performance reached a plateau on Day 1 (Group 2 and Group 4). Our results, together with previous studies, suggest that saturation learning induces consolidation of the motor memory regardless of the task type.

The motor memory that could be consolidated by saturation learning might correspond to the internal model in the framework of the motor computational theory. Shadmehr and his colleagues proposed that a novel internal model is maintained in the working memory; it not only persists for a short time but also easily suffers from interference by other new learning in the initial stage of learning [16, 56]. In addition, an unstable internal model requires further practice to be transferred to long-term memory [16, 57]. The inference from our results does not contradict these assumptions.

The second hypothesis, i.e., a longer time interval between practice sessions prevents the new internal model from being activated, was not supported. The analysis of aftereffects on Day 2 revealed that there was neither a significant interaction between the time interval between practice sessions and the day of practice nor a significant main effect of the time interval. Thus, the amplitude of the aftereffects did not differ between the 24-h-interval condition and the 48-h-interval condition. Only one study simultaneously investigated the relationship between aftereffects and the time interval between practice sessions [9], which is discussed later in detail. Although further investigation is necessary to reveal the relationship, our results at least suggest that aftereffects, which reflects the acquisition of a new internal model, might not decay from 24 h to 48 h. The amount of practice rather than the time interval between practice sessions seems to affect the consolidation and retention of a new internal model.

### Savings and amount of practice and time interval between practice sessions

Based on the idea that savings reflects the process of reacquisition or re-expression of an internal model learned once [4, 40, 58–61], the present results suggest that the reacquisition or re-expression of a new internal model is facilitated as the amount of practice increases; the larger savings in the 20-session practice groups (Group 2 and Group 4) supports the first hypothesis that a new internal model is stabilized by additional practice after task performance reaches a plateau. In contrast, several studies have argued that savings is not related to the state of internal models but emerges on the basis of model-free processes such as use-dependent plasticity and operant reinforcement [12, 35, 36, 62]; movements that successfully achieved the goal of the task are reinforced, the motor memory is recalled, and the learning efficiency is improved. In terms of this mechanism, as extensive practice after the task performance reaches a plateau brings a sequence of successes to the participants, the present results can also be interpreted as re-learning on Day 2 being facilitated by operant reinforcement.

The present result that savings was not affected by the time interval between practice sessions can be interpreted in terms of both internal models and reinforced memory; if we assume that savings reflects the process of reacquisition or re-expression of a new internal model [4, 40, 58–61], the new model that is acquired once would not be eliminated with the passage of time. Similarly, if we assume that savings reflect the recall of motor memory that was reinforced by a model-free mechanism [12, 35, 36], the reinforced motor memory would not decay with the passage of time.

In any case, our results are not consistent with those of a previous study that used the same time intervals as those in our experiment [8]. Krakauer, Ghez [8] reported that regardless of the amount of practice, greater savings was observed in the experimental groups where the participants performed a re-learning task 24 h after the first learning session than in the groups with a 48-h interval between two learning sessions (Experiment 4). The discrepancy between the results of Krakauer, Ghez [8] and our results might be explained if we assume that motor learning in the participants in the present study was insufficient in the 10-session groups; if learning in the 10-session groups was less, the magnitude of savings would also be less in these groups than in the 20-session groups. Motor learning could be insufficient owing to the difference between the tasks involved in the studies; Krakauer, Ghez [8] used a reaching task and we used a tracking task. However, further investigation is required as it is difficult to discuss these possibilities in detail.

### Comparison with results involving a reaching task

Here, we compare the results of Trempe and Proteau [9] with our results in detail, as both studies manipulated the amount of practice and the length of time interval between practice sessions to investigate the effect of these factors on motor learning. The major difference between the studies was the type of motor learning tasks; Trempe and Proteau [9] used a reaching task while the present study employed a tracking task. In addition, they set the amount of practice on Day 1 at 24 trials or 144 trials and the time interval at 10 min or 24 h, whereas we set the amount of practice at 10 sessions (40 trials) or 20 sessions (80 trials) and the time interval at 24 h or 48 h. Though there were several differences in the experimental paradigm between Trempe and Proteau [9] and the present study, the main conclusions were consistent, i.e., the condition with the greater amount of practice showed greater aftereffects.

In terms of off-line learning, while the gain in learning was significantly greater in the 24-h-interval groups than in the 10-min-interval groups in the work of Trempe and Proteau [9], there was no statistical significance either in the main effects of the amount of practice and the time interval between practice sessions or the interaction between them. The discrepancy between the present study and that of Trempe and Proteau [9] could be explained by the effect of sleep; in the work of Trempe and Proteau [9], one condition involved sleep but the other did not after the first learning session (10 min vs. 24 h). By contrast, both conditions in our study involved night sleep despite the difference in how many times the participants slept. Previous studies have revealed that sleep considerably enhances off-line learning [63–65], the amount of which largely depends on the types of learning and of learning measures [44, 66]. The discrepancy may be explained by assuming that the second-time sleep would not facilitate further learning. In general, with minor differences owing to the experimental condition, the results of Trempe and Proteau [9] are consistent with our results, and they suggest that a tracking task and a reaching task have a common underlying mechanism of aftereffects and off-line learning of visuomotor rotation.

### Advantages and limitations of the present study

The findings from studies involving the tracking task strengthens the findings from previous studies on human motor learning. First, as the tracking task requires the participants to move their arm continuously for a longer time than the reaching task [24, 43–45, 55], the tracking task prevents participants from adopting explicit strategies to achieve the goal of the task [55]. Second, it requires the participants to pay attention to the effect of their physical movement in an external environment rather than to their own body, and the external attention leads to the achievement of efficient motor learning [67–69]. Third, the tracking task consists of a succession of reaching movements in various directions, and it would accordingly facilitate the generalization of learning [70]. Finally, the tracking task likely duplicates motor learning in daily or clinical situations that is fluid and requires continuous responses. As there are relatively few visuomotor adaptation studies involving the tracking task, further investigation using the task is required to elucidate the mechanism of human motor learning along with the findings from previous studies involving the reaching task.

The present study has the following limitations. First, only a few conditions of the amount of practice (10 sessions and 20 sessions) and the time interval between practice sessions (24 h and 48 h) were considered. With the present experimental paradigm, we did not investigate motor learning for the duration which is actually required in real-life situations such as physical therapy and athletic training scenarios. Second, the lack of conclusive evidence of a learning plateau is a significant limitation of the present study. It is difficult to identify a learning plateau using statistical methods, particularly in a relatively short-term experiment. However, note that it is not important that a learning plateau was achieved at the 5th and 6th sessions in the 10-session groups (Groups 1 and 3). The assumption that the errors at the end of the rotation sessions did not largely decreased was, at least, not statistically disproved (that is, null result), although it is not direct evidence for supporting the assumption. Finally, the effect of a contextual cue in the tracking task of our study should be considered. In the present study, different colors of the target in the tracking task could play the role of a contextual cue regarding perturbation. Several previous studies have revealed that color contextual cues in a motor learning task facilitate the switching of multiple internal models [71–74]. In addition, Taylor, Krakauer [51] reported that explicit cuing hinders aftereffects in a visuomotor rotation task. Taking these findings into account, there is a possibility that the different colors of the target in the tracking task in the present study could be contextual cues that decay the aftereffect. In the present study, the aftereffects could be less than observed in previous studies because of the different colors of the target in the tracking task.

## Conclusion

The present study aimed to reveal the relationship between the amount of practice and time interval between practice sessions and the retention and consolidation of a new internal model for efficient motor learning. Two main suggestions were provided. First, additional practice after reaching a task performance plateau is important for the consolidation of a new internal model. Second, two-day rest does not negatively affect the learning of a simple visuomotor task. We emphasize here that the present study provided suggestions consistent with those of previous studies regardless of the type of motor learning task. The suggestions of this study might eliminate the extra time and labor required for motor learning, streamline rehabilitation in clinical settings, and facilitate efficient sports training.

## Supporting information

S1 File The dataset for the experiment. Each sheet contains the dataset for a paired *t*- tests for learning plateau, ANOVA for aftereffects and savings respectively.
(XLSX)

## References

[1] Borresen J, Lambert MI. The quantification of training load, the training response and the effect on performance. Sports medicine. 2009;39(9):779–95. 10.2165/11317780-000000000-00000 19691366

[2] Magill RA, Hall KG. A review of the contextual interference effect in motor skill acquisition. Human movement science. 1990;9(3–5):241–89.

[3] Schmidt RA, Bjork RA. New conceptualizations of practice: Common principles in three paradigms suggest new concepts for training. Psychological science. 1992;3(4):207–18.

[4] Guadagnoli MA, Lee TD. Challenge point: a framework for conceptualizing the effects of various practice conditions in motor learning. Journal of motor behavior. 2004;36(2):212–24. 10.3200/JMBR.36.2.212-224 15130871

[5] Poolton J, Masters R, Maxwell J. Passing thoughts on the evolutionary stability of implicit motor behaviour: Performance retention under physiological fatigue. Consciousness and cognition. 2007;16(2):456–68. 10.1016/j.concog.2006.06.008 16876433

[6] Yin HH, Mulcare SP, Hilário MR, Clouse E, Holloway T, Davis MI, et al Dynamic reorganization of striatal circuits during the acquisition and consolidation of a skill. Nature neuroscience. 2009;12(3):333 10.1038/nn.2261 19198605 PMC2774785

[7] Karni A, Meyer G, Rey-Hipolito C, Jezzard P, Adams MM, Turner R, et al The acquisition of skilled motor performance: fast and slow experience-driven changes in primary motor cortex. Proceedings of the National Academy of Sciences. 1998;95(3):861–8. 10.1073/pnas.95.3.861 PMC33809 9448252

[8] Krakauer JW, Ghez C, Ghilardi MF. Adaptation to visuomotor transformations: consolidation, interference, and forgetting. Journal of Neuroscience. 2005;25(2):473–8. 10.1523/JNEUROSCI.4218-04.2005 15647491 PMC6725486

[9] Trempe M, Proteau L. Distinct consolidation outcomes in a visuomotor adaptation task: Off-line leaning and persistent after-effect. Brain and cognition. 2010;73(2):135–45. 10.1016/j.bandc.2010.04.005 20488608

[10] Trempe M, Sabourin M, Proteau L. Success modulates consolidation of a visuomotor adaptation task. Journal of Experimental Psychology: Learning, Memory, and Cognition. 2012;38(1):52 10.1037/a0024883 21843020

[11] Tanaka H, Krakauer JW, Sejnowski TJ. Generalization and multirate models of motor adaptation. Neural computation. 2012;24(4):939–66. 10.1162/NECO_a_00262 22295980 PMC3420803

[12] Kitago T, Ryan SL, Mazzoni P, Krakauer JW, Haith AM. Unlearning versus savings in visuomotor adaptation: comparing effects of washout, passage of time, and removal of errors on motor memory. Frontiers in human neuroscience. 2013;7:307 10.3389/fnhum.2013.00307 23874277 PMC3711055

[13] Nettersheim A, Hallschmid M, Born J, Diekelmann S. The role of sleep in motor sequence consolidation: stabilization rather than enhancement. Journal of Neuroscience. 2015;35(17):6696–702. 10.1523/JNEUROSCI.1236-14.2015 25926448 PMC4412892

[14] Criscimagna-Hemminger SE, Shadmehr R. Consolidation patterns of human motor memory. Journal of Neuroscience. 2008;28(39):9610–8. 10.1523/JNEUROSCI.3071-08.2008 18815247 PMC2665258

[15] Pekny SE, Shadmehr R. Optimizing effort: increased efficiency of motor memory with time away from practice. Journal of neurophysiology. 2014;113(2):445–54. 10.1152/jn.00638.2014 25355964 PMC4297790

[16] Shadmehr R, Brashers-Krug T. Functional stages in the formation of human long-term motor memory. Journal of Neuroscience. 1997;17(1):409–19. 8987766 10.1523/JNEUROSCI.17-01-00409.1997 PMC6793707

[17] Kawato M, Furukawa K, Suzuki R. A hierarchical neural-network model for control and learning of voluntary movement. Biological cybernetics. 1987;57(3):169–85. 3676355 10.1007/BF00364149

[18] Schweighofer N, Arbib MA, Kawato M. Role of the cerebellum in reaching movements in humans. I. Distributed inverse dynamics control. European Journal of Neuroscience. 1998;10(1):86–94. 9753116 10.1046/j.1460-9568.1998.00006.x

[19] Wolpert DM, Miall RC, Kawato M. Internal models in the cerebellum. Trends in cognitive sciences. 1998;2(9):338–47. 21227230 10.1016/s1364-6613(98)01221-2

[20] Kawato M. Internal models for motor control and trajectory planning. Current opinion in neurobiology. 1999;9(6):718–27. 10607637 10.1016/s0959-4388(99)00028-8

[21] Miall RC, Wolpert DM. Forward models for physiological motor control. Neural networks. 1996;9(8):1265–79. 12662535 10.1016/s0893-6080(96)00035-4

[22] Desmurget M, Grafton S. Forward modeling allows feedback control for fast reaching movements. Trends in cognitive sciences. 2000;4(11):423–31. 11058820 10.1016/s1364-6613(00)01537-0

[23] Kawato M, Gomi H. A computational model of four regions of the cerebellum based on feedback-error learning. Biological cybernetics. 1992;68(2):95–103. 1486143 10.1007/BF00201431

[24] Imamizu H, Miyauchi S, Tamada T, Sasaki Y, Takino R, PuÈtz B, et al Human cerebellar activity reflecting an acquired internal model of a new tool. Nature. 2000;403(6766):192 10.1038/35003194 10646603

[25] Blakemore S-J, Frith CD, Wolpert DM. The cerebellum is involved in predicting the sensory consequences of action. Neuroreport. 2001;12(9):1879–84. 11435916 10.1097/00001756-200107030-00023

[26] Blakemore S-J, Sirigu A. Action prediction in the cerebellum and in the parietal lobe. Experimental Brain Research. 2003;153(2):239–45. 10.1007/s00221-003-1597-z 12955381

[27] Buch ER, Young S, Contreras-Vidal JL. Visuomotor adaptation in normal aging. Learning & memory. 2003;10(1):55–63. 12551964 10.1101/lm.50303 PMC196655

[28] Krakauer JW. Motor learning and consolidation: the case of visuomotor rotation. Progress in motor control: Springer; 2009 p. 405–21. 10.1007/978-0-387-77064-2_21 PMC2672910 19227512

[29] Kravitz JH, Yaffe F. Conditioned adaptation to prismatic displacement with a tone as the conditional stimulus. Perception & Psychophysics. 1972;12(3):305–8.

[30] Bock O. Components of sensorimotor adaptation in young and elderly subjects. Experimental Brain Research. 2005;160(2):259–63. 10.1007/s00221-004-2133-5 15565436

[31] Mazzoni P, Krakauer JW. An implicit plan overrides an explicit strategy during visuomotor adaptation. Journal of neuroscience. 2006;26(14):3642–5. 16597717 10.1523/JNEUROSCI.5317-05.2006 PMC6674132

[32] Shadmehr R, Mussa-Ivaldi FA. Adaptive representation of dynamics during learning of a motor task. Journal of Neuroscience. 1994;14(5):3208–24. 8182467 10.1523/JNEUROSCI.14-05-03208.1994 PMC6577492

[33] Ostry DJ, Feldman AG. A critical evaluation of the force control hypothesis in motor control. Experimental brain research. 2003;153(3):275–88. 10.1007/s00221-003-1624-0 14610628

[34] Zarahn E, Weston GD, Liang J, Mazzoni P, Krakauer JW. Explaining savings for visuomotor adaptation: linear time-invariant state-space models are not sufficient. Journal of neurophysiology. 2008;100(5):2537–48. 10.1152/jn.90529.2008 18596178 PMC2585408

[35] Haith AM, Huberdeau DM, Krakauer JW. The influence of movement preparation time on the expression of visuomotor learning and savings. Journal of neuroscience. 2015;35(13):5109–17. 10.1523/JNEUROSCI.3869-14.2015 25834038 PMC6705405

[36] Huang VS, Haith A, Mazzoni P, Krakauer JW. Rethinking motor learning and savings in adaptation paradigms: model-free memory for successful actions combines with internal models. Neuron. 2011;70(4):787–801. 10.1016/j.neuron.2011.04.012 21609832 PMC3134523

[37] Krakauer JW, Shadmehr R. Consolidation of motor memory. Trends in neurosciences. 2006;29(1):58–64. 10.1016/j.tins.2005.10.003 16290273 PMC2553888

[38] Ghilardi M-F, Ghez C, Dhawan V, Moeller J, Mentis M, Nakamura T, et al Patterns of regional brain activation associated with different forms of motor learning. Brain research. 2000;871(1):127–45. 10882792 10.1016/s0006-8993(00)02365-9

[39] Krakauer JW, Pine ZM, Ghilardi M-F, Ghez C. Learning of visuomotor transformations for vectorial planning of reaching trajectories. Journal of Neuroscience. 2000;20(23):8916–24. 11102502 10.1523/JNEUROSCI.20-23-08916.2000 PMC6773094

[40] Smith MA, Ghazizadeh A, Shadmehr R. Interacting adaptive processes with different timescales underlie short-term motor learning. PLoS biology. 2006;4(6):e179 10.1371/journal.pbio.0040179 16700627 PMC1463025

[41] Heuer H, Hegele M. Adaptation to visuomotor rotations in younger and older adults. Psychology and aging. 2008;23(1):190 10.1037/0882-7974.23.1.190 18361666

[42] Morehead JR, Qasim SE, Crossley MJ, Ivry R. Savings upon re-aiming in visuomotor adaptation. Journal of neuroscience. 2015;35(42):14386–96. 10.1523/JNEUROSCI.1046-15.2015 26490874 PMC4683692

[43] Itaguchi Y, Fukuzawa K. Adaptive changes in automatic motor responses based on acquired visuomotor correspondence. Exp Brain Res. 2019:1–13. Epub 2018/10/27. 10.1007/s00221-018-5409-x . 30361773

[44] Kaida K, Itaguchi Y, Iwaki S. Interactive effects of visuomotor perturbation and an afternoon nap on performance and the flow experience. PloS one. 2017;12(2):e0171907 10.1371/journal.pone.0171907 28182742 PMC5300137

[45] Künzell S, Sießmeir D, Ewolds H. Validation of the continuous tracking paradigm for studying implicit motor learning. Experimental psychology. 2017. 10.1027/1618-3169/a000343 28059029

[46] Ewolds HE, Bröker L, De Oliveira RF, Raab M, Künzell S. Implicit and explicit knowledge both improve dual task performance in a continuous pursuit tracking task. Frontiers in psychology. 2017;8:2241 10.3389/fpsyg.2017.02241 29312083 PMC5744266

[47] Bond KM, Taylor JA. Flexible explicit but rigid implicit learning in a visuomotor adaptation task. American Journal of Physiology-Heart and Circulatory Physiology. 2015. 10.1152/jn.00009.2015 PMC4473515 25855690

[48] Morehead JR, Taylor JA, Parvin DE, Ivry RB. Characteristics of implicit sensorimotor adaptation revealed by task-irrelevant clamped feedback. Journal of cognitive neuroscience. 2017;29(6):1061–74. 10.1162/jocn_a_01108 28195523 PMC5505262

[49] Kim S, Oh Y, Schweighofer N. Between-trial forgetting due to interference and time in motor adaptation. PloS one. 2015;10(11):e0142963 10.1371/journal.pone.0142963 26599075 PMC4657926

[50] Neville K-M, Cressman EK. The influence of awareness on explicit and implicit contributions to visuomotor adaptation over time. Experimental brain research. 2018;236(7):2047–59. 10.1007/s00221-018-5282-7 29744566

[51] Taylor JA, Krakauer JW, Ivry RB. Explicit and implicit contributions to learning in a sensorimotor adaptation task. Journal of Neuroscience. 2014;34(8):3023–32. 10.1523/JNEUROSCI.3619-13.2014 24553942 PMC3931506

[52] Brainard DH, Vision S. The psychophysics toolbox. Spatial vision. 1997;10:433–6. 9176952

[53] Pelli DG. The VideoToolbox software for visual psychophysics: Transforming numbers into movies. Spatial vision. 1997;10(4):437–42. 9176953

[54] Yin P-B, Kitazawa S. Long-lasting aftereffects of prism adaptation in the monkey. Experimental brain research. 2001;141(2):250–3. 10.1007/s002210100892 11713636

[55] Cunningham H, Welch RB. Multiple concurrent visual-motor mappings: implications for models of adaptation. Journal of Experimental Psychology: Human Perception and Performance. 1994;20(5):987 7964533 10.1037//0096-1523.20.5.987

[56] Wigmore V, Tong C, Flanagan JR JJoEPHP, Performance. Visuomotor rotations of varying size and direction compete for a single internal model in a motor working memory. 2002;28(2):447. 10.1037//0096-1523.28.2.447 11999865

[57] Brashers-Krug T, Shadmehr R, Bizzi E. Consolidation in human motor memory. Nature. 1996;382(6588):252 10.1038/382252a0 8717039

[58] Haruno M, Wolpert DM, Kawato M. Mosaic model for sensorimotor learning and control. Neural computation. 2001;13(10):2201–20. 10.1162/089976601750541778 11570996

[59] Kording KP, Tenenbaum JB, Shadmehr R. The dynamics of memory as a consequence of optimal adaptation to a changing body. Nature neuroscience. 2007;10(6):779 10.1038/nn1901 17496891 PMC2551734

[60] Lee J-Y, Schweighofer N. Dual adaptation supports a parallel architecture of motor memory. Journal of Neuroscience. 2009;29(33):10396–404. 10.1523/JNEUROSCI.1294-09.2009 19692614 PMC2789989

[61] Ajemian R, D’Ausilio A, Moorman H, Bizzi E. Why professional athletes need a prolonged period of warm-up and other peculiarities of human motor learning. Journal of motor behavior. 2010;42(6):381–8. 10.1080/00222895.2010.528262 21184356

[62] Huberdeau DM, Krakauer JW, Haith AM. Dual-process decomposition in human sensorimotor adaptation. Current Opinion in Neurobiology. 2015;33:71–7. 10.1016/j.conb.2015.03.003 25827272

[63] Walker MP, Brakefield T, Morgan A, Hobson JA, Stickgold R. Practice with sleep makes perfect: sleep-dependent motor skill learning. Neuron. 2002;35(1):205–11. 12123620 10.1016/s0896-6273(02)00746-8

[64] Albouy G, Ruby P, Phillips C, Luxen A, Peigneux P, Maquet P. Implicit oculomotor sequence learning in humans: Time course of offline processing. Brain research. 2006;1090(1):163–71. 10.1016/j.brainres.2006.03.076 16677617

[65] Doyon J, Bellec P, Amsel R, Penhune V, Monchi O, Carrier J, et al Contributions of the basal ganglia and functionally related brain structures to motor learning. Behavioural brain research. 2009;199(1):61–75. 10.1016/j.bbr.2008.11.012 19061920

[66] Itaguchi Y, Kaneko F. Motor priming by movement observation with contralateral concurrent action execution. Human movement science. 2018;57:94–102. 10.1016/j.humov.2017.11.007 29195131

[67] Wulf G, Prinz W. Directing attention to movement effects enhances learning: A review. Psychonomic bulletin & review. 2001;8(4):648–60. 11848583 10.3758/bf03196201

[68] Wulf G, Lewthwaite R. Effortless motor learning? An external focus of attention enhances movement effectiveness and efficiency. Effortless attention: A new perspective in attention and action. 2010:75–101.

[69] Wulf G, Shea CH. Principles derived from the study of simple skills do not generalize to complex skill learning. Psychonomic bulletin & review. 2002;9(2):185–211. 12120783 10.3758/bf03196276

[70] Neva JL, Henriques DY. Visuomotor adaptation and generalization with repeated and varied training. Experimental brain research. 2013;226(3):363–72. 10.1007/s00221-013-3444-1 23455723

[71] Osu R, Hirai S, Yoshioka T, Kawato M. Random presentation enables subjects to adapt to two opposing forces on the hand. Nature neuroscience. 2004;7(2):111 10.1038/nn1184 14745452

[72] Wada Y, Kawabata Y, Kotosaka S, Yamamoto K, Kitazawa S, Kawato M. Acquisition and contextual switching of multiple internal models for different viscous force fields. Neuroscience research. 2003;46(3):319–31. 12804793 10.1016/s0168-0102(03)00094-4

[73] Hinder MR, Tresilian JR, Riek S, Carson RG. The contribution of visual feedback to visuomotor adaptation: how much and when? Brain research. 2008;1197:123–34. 10.1016/j.brainres.2007.12.067 18241844

[74] Addou T, Krouchev NI, Kalaska JF. Colored context cues can facilitate the ability to learn and to switch between multiple dynamical force fields. American Journal of Physiology-Heart and Circulatory Physiology. 2011. 10.1152/jn.00869.2010 21490278

